# Improving Teenagers’ Divergent Thinking With Improvisational Theater

**DOI:** 10.3389/fpsyg.2018.01759

**Published:** 2018-09-25

**Authors:** Mathieu Hainselin, Alexandre Aubry, Béatrice Bourdin

**Affiliations:** CRP-CPO, EA 7273, Université de Picardie Jules Verne, Amiens, France

**Keywords:** improv, divergent thinking, pedagogy, school, embodiment, action

## Abstract

Improvisational theater (improv) is supposed to have an impact on cognitive processes (divergent thinking, flexibility, language, memory, problem solving, and co-construction), academic performance, and everyday life in many ways. However, little research studied on the psychological impact of improv, with some results highlighting a divergent thinking enhancement in children and adults, but not with teenagers, one of the most important age groups to practice improv. Therefore, this study aims to assess divergent thinking for middle school students before and after an 11-weeks session compared to a control group with a sport practice. The Alternative Uses Task (AUT) was used before and after the session for both groups to evaluate divergent thinking. The improv group had better performance in originality, flexibility and gave less prototypical items after the improv sessions compared to before, while the control group performance was similar before and after. Our results suggest that improv helps teenagers’ divergent thinking to improve, not only with experimental games in the lab context but also after ecological sessions. We urge scientists to study in depth psychological impacts of improvisational theater and applied improvisation, for a better understanding of improv and as a model to study embodied cognition.

## Introduction

In a fast changing century, many people in arts, science, teaching, management, or technology wonder how to innovate and produce novel and useful resources, which are the most widely accepted elements regarding the definition of creativity (Sternberg and Lubart, 1993; Ritter and Mostert, 2017). Considering this necessity to develop good creativity skills, many people turn to arts, mostly theater and music. In this context, the success of improvisational theater (improv), supposed to enhance creativity (Bermant, 2013), spreads into various areas beyond theater (i.e., applied improvisation): medical doctors, psychologists, teachers, students, managers, negotiators and scientists attend improv workshops (Bermant, 2013; Bernstein, 2014; Hainselin et al., 2017b; Hoffmann-Longtin et al., 2017). The effects of improv on creativity, claimed by improv teacher, have not yet been studied extensively in cognitive sciences. The most of these studies used some improv games but not a full cycle usually used in improv training (Lewis and Lovatt, 2013; Sowden et al., 2015). The current project aims to focus on the impact of an ecological improv cycle on teenagers, which have never been studied, in our knowledge. In the remaining part of the introduction, we will focus on one aspect of creativity (divergent thinking) and psychological impact of improvisation.

### Divergent Thinking

Classic creativity models include the implication of associative processes (Megalakaki et al., 2012). Lubart (2001, p. 295) specified the cognitive processes involved in creativity as “a capacity to produce many ideas (fluency), an ability to change one’s mental set (flexibility), an ability to reorganize, an ability to deal with complexity, and an ability to evaluate.” Creativity includes divergent thinking and convergent thinking. Divergent thinking supposed to generate multiple different answers or to think “out of the box.” One of its most common evaluations is the generation of alternative ideas, as assessed in the Alternative Uses Task (AUT; Guilford, 1967). Convergent thinking is the ability to find the most coherent idea of several answers. This knowledge dependent “evaluation of the novelty” interacts with divergent thinking and is “most effective in situations where a ready-made answer exists and need simply to be recalled from stored information” (Cropley, 2006). While it is commonly admitted that creativity is both divergent and convergent thinking, the latter is sometimes referred to as “uncreative” (Ritter and Ferguson, 2017) and previous research pinpointed that divergent thinking is a widely accepted measure of creativity (Runco and Acar, 2012; Kenett et al., 2014). Mumford’s team (1991) proposed a model highlighting eight core processing activities for creative efforts: (1) problem definition, (2) information encoding, (3) category search, (4) specification of best-fitting categories, (5) combination and reorganization of best-fitting categories, (6) idea evaluation, (7) implementation, and (8) monitoring. Although, these eight processes should be taken into account for creativity assessment, Mumford et al. (2008), in a Silvia’s et al. (2008) team commentary paper, wrote that the idea generation measure used in this latter study is consistent with the traditional approach in the literature, probably for feasibility issues. Mumford et al. (1991) suggest that this kind of output-based measure might mostly reflect the idea generation process and not all of the eight they highlighted. While we believe research should focus on assessing these eight core processing activities, we also know that it is very time consuming and not always possible in ecological evaluation, especially with children and teenagers.

Many psychological factors, cognitive and emotional, can influence divergent thinking (for review and full description of cognitive processes involved, see Megalakaki et al., 2012). In its early research, Guilford scored his task for flexibility (changing from one idea to the others) and fluency (producing different ideas), two dimensions widely highlighted in the literature in divergent thinking assessments. Heuristics and information processing (including the associative processes of binding) also influence divergent thinking; they can be related to associative and executive processes (i.e., updating, switching, inhibition; Beaty et al., 2014). The role of associative processes, as well as executive control, seems to be particularly important to divergent thinking (Forthmann et al., 2016). Indeed, connecting the dots in a particular way, due to good associative process, might lead to see patterns where others cannot; in other words, it can be the combination of remote associations into new and useful combinations (Kenett et al., 2014). The associative processes have strong connections with memory to help creativity processes to emerge (for extended discussion, see Jung and Vartanian, 2018). Personality factors, notably openness, risk taking and perseverance also have an impact on divergent thinking (Lubart et al., 2009).

### Improvisational Theater

Improvisational theater (improv) is a specific theater form in which the performance is spontaneous (i.e., without previous scenario written nor prepared). In this condition, going on stage without a single prepared word, costume or décor requires risk taking and perseverance to keep falling and getting up for every performance (Johnstone, 1999; Bermant, 2013). Hoffmann-Longtin et al. (2017) recently pinpointed that rather being innately spontaneous, “professional improvisers develop the ability to listen closely, focus, accept others’ ideas and support one another through improvisation games.” In its improv manual, Tournier (2001) highlights 10 values including to listen, accept, build, innovate and dare to. In other words, we can argue that improv is supposed to develop processes that can be referred to as executive functions (such as flexibility and fluency), information processing (including binding), in addition to risk taking and perseverance. Although, there is very few psychological science papers on cognitive processes involved in improv, we can highlight that these processes are very similar to those involved in divergent thinking. Beyond this obvious overlap, previous improv papers involved cognitive evaluation.

### Cognitive Impact of Improvisation

Improv teachers, learners, and scientists involved in improv (Bermant, 2013; Bernstein, 2014) or jazz improvisation (Doyle, 2017) usually demonstrate higher levels of creativity, memory, well-being, and less anxiety, all of which may be possible improv benefits (Bermant, 2013). However, there is little scientific evidence for these supposed benefits.

The emotional impact of improv was only very recently assessed to find it can help reduce anxiety and depression in young and older (27–72 years old) adults (Krueger et al., 2017). At our knowledge, only one study assessed the impact of improv on memory and showed a better ability to remember a dramatic text when played in an improvisation scene condition compared to reading only or writing about the scene or group discussions conditions (Scott et al., 2001). This result is consistent with the enactment effect (i.e., better memory for performed actions than for verbally encoded action sentences) and the embodied nature of improv (Borghi and Caruana, 2015; Hainselin et al., 2017a). In its history, cognitive and physical dimensions of improv were always very important, including its name. In the 1970’s, it was difficult to define improv as an alternative education tool, a theater or sport practice, leading to refer to as theatresport (Johnstone, 1999). Most of the studies used improv exercises thought to enhance divergent thinking, as free walking (Kuo and Yeh, 2016), in jazz music (Benedek et al., 2014), spontaneous sentences and conversation (Lewis and Lovatt, 2013) or gesture while speaking (Lewis et al., 2015). If there is some evidence that improv increases divergent thinking during adulthood, these researches used laboratory situation with specific exercises, very different from the standard improv courses. Moreover, they did not use a physical activity control group to distinguish between the divergent thinking due to specific improv courses and physical activity’s effect. Improv games for elementary school children found similar impact on divergent thinking than for adults (Sowden et al., 2015). In both studies with adults (Lewis and Lovatt, 2013) and children (Sowden et al., 2015), participants only took part in a short session of improv games, but no study assessed the impact of a more ecological improv program with multiple sessions of 1–2 h.

Thus, this study aimed to evaluate the impact of improv on teenagers’ divergent thinking, following a standard course and if it differed from a sports control group. Adolescence is a critical period for cognitive development; there are many improv courses for teenagers in middle school but, as far we know, no scientific study on its impact yet.

## Materials and Methods

### Participants

A total of 35 participants took part in this study; three of them were not included because of missing data or stopping an activity before the end; we also excluded 2 teenagers who skipped a grade to avoid age or intelligence differences. We included 18 participants (9 males and 9 females) for the improv group and 12 (8 males and 4 females) for the control group; all of them were seventh graders. There was no significant age (improv group mean = 11.39; *SD* = 0.50; control group mean = 11.75; *SD* = 0.62) [*t*(20.166) = 1.681, *p* = 0.108] nor gender [χ^2^(1) = 0.814, *p* < 0.367] differences between the groups. All of them were French speakers. All teenagers gave their written consent to be part of the research and we had approval from their parents, headmasters and teachers. None of them had neurological or psychiatric history, nor developmental learning disorder.

### Materials

The AUT is a task based on Guilford work (1967). Participants had to list as many possible uses for common items. We had the same methodology than Lewis and Lowatt paper (2013): the two items were a paperclip and a remote control and participants had 3 min to list alternative uses for each item. Scoring comprised of five components:

(1) Prototypical items: Number of prototypical response. For paperclip, prototypical response would be “holding sheets of paper together” and for remote control “turn the volume down” or “change the channel.” The other four components are classical for AUT, but we added this fifth after observing 72% of our participants gave at least one prototypical use. In previous divergent thinking studies with similar task, it has previously been taken into account as “standard actions” (Harrington et al., 1983) or “usual or popular responses” (Milgram et al., 1978).
(2) Originality: How original each response is. We compared each response to every responses regarding one item. Reponses with over 5% rate are considered common (0 point), between 1 and 5% unusual (1 point) and <1% ones as unique (2 points). The cumulated score of all given items for each item is the originality score. The more original the responses are, the higher the score is.
(3) Fluency: Number of different responses for each item.
(4) Flexibility: Number of different categories for each item. For example, if a participant wrote “necklace, ring and earring” as alternative uses for the paperclip, the flexibility score is 1, as there is one category (jewelry). For a participant who wrote “necklace, key and Christmas’ tree decoration,” the flexibility score would be 3.
(5) Elaboration: Amount of details. Each detail adds one point. For example, “a key” is 0 point but “a key to open the door which is always locked” is 2 points: 1 for the door opening and 1 for the always locked detail.

Considering the influence of fluency on originality (easier to have original answers within 20 propositions than in 5) and flexibility (more categories can be found within more answers), and regarding methodological recommendations (Clark and Mirels, 1970; Mouchiroud and Lubart, 2001), we calculated a ratio score by dividing prototypical items, originality and flexibility scores by the fluency score.

### Procedure

All participants took the AUT during the week preceding the first improv course for the pre-assessment and during the week after the last improv course for the post-assessment evaluation.

The 11 improvisation sessions took place in the teenagers’ school, during lunchtime for 60 min session. Each session theme is described in **Table 1**. The control group participants were enrolled in a sport session for the same amount of time and frequency.

**Table 1:** Improv sessions description.

Session number	Theme	Example of improv games
Session 1	Presentation, introductive improv games	Zip Zap Zop: people pass the energy across the circle (in the form of a Zip, a Zap, or a Zop), they make eye contact with the person they send the energy to
Session 2	Co-construction	Listening to one other improviser’s idea and add a detail to make this idea richer
Session 3	Emotions	Turning the emotion volume from 1 (a tiny smile) to 5 (being over the moon)
Session 4	Saying yes	Playing a scene without saying no to the other people idea, then saying “yes and” to it
Session 5	Characters	An imposed character is given to the improviser who has to play it (i.e., man on the moon, tired pupil)
Session 6	Reactivity	All participants had to rank themselves regarding different criteria (i.e., shoe size) within 20 s
Session 7	Improv categories	Categories included playing without moving or dubbing (one moves his/her lips while the other speaks)
Session 8	Listening	Walking with the eyes closed, only guided by another improviser’s voice
Session 9	Imagination	Describe a world where you have never been previously
Session 10	Show preparation 1	Includes all previous sessions themes
Session 11	Show preparation 2	Includes all previous sessions themes


### Statistical Methodology

All data were analyzed with R 3.4.3 (R Core Team, 2017). All statistical analyses were, respectively, realized on the number of fluency and the ratio score of prototypical, flexibility, originality and elaboration items. We carried out each statistical analysis using repeated measured ANOVA with Group (Improv, Control) as between participants’ factor and Condition (Pre-assessment, Post-assessment) as the within-participants factor.

All repeated measured ANOVA met the assumption of sphericity. When the assumption of normality of residuals was violated, the rank transformation was realized on the dependent variables (Zimmerman and Zumbo, 1993). The use of transformation was indicated in the statistical analysis.

The data and R script to replicate all statistical analyses are available on the Open Science Framework (OSF) platform at https://osf.io/ysxer/. Data are avalaible in **Supplementary Material** as well.

## Results

Regarding statistical methodology, we present rank transformed data for the ratio score of elaboration and number of fluency, but non-transformed data for the ratio score of prototypical items, originality and flexibility. In summary, for all analyses (Elaboration, Prototypical items, Originality, and Flexibility) except for Fluency, we used ratio scores. However, raw data are available in **Table 2** for readers who want the information.

**Table 2:** Raw data, means, and standard deviation for each condition and group.

Index	Score	Improv (*n* = 18)			Control (*n* = 12)		
		*M* (*SD*)	Skewness	Kurtosis	*M* (*SD*)	Skewness	Kurtosis
**Pre-assessment**					
Fluency	Raw	4.111 (1.632)	0.360	–0.386	4.167 (1.249)	–0.508	–0.753
Prototypical	Raw	1.694 (1.426)	0.591	–0.639	0.958 (1.076)	0.952	–0.095
	Ratio	0.439 (0.320)	0.038	–1.399	0.274 (0.311)	0.917	–0.193
Originality	Raw	2.944 (3.038)	1.550	1.523	3.667 (2.462)	0.210	–1.564
	Ratio	0.691 (0.498)	0.342	–1.316	0.822 (0.434)	0.177	–1.304
Flexibility	Raw	2.111 (1.844)	1.556	1.894	2.958 (1.544)	–0.302	–1.753
	Ratio	0.478 (0.273)	0.398	–0.698	0.682 (0.249)	–0.562	–0.557
Elaboration	Raw	1.000 (0.728)	0.541	–0.324	1.583 (1.104)	0.132	–1.244
	Ratio	0.290 (0.267)	1.103	0.540	0.262 (0.404)	–0.114	–1.325
**Post-assessment**					
Fluency	Raw	6.222 (3.878)	1.397	2.102	4.458 (1.437)	–0.492	–1.004
Prototypical	Raw	0.472 (0.866)	2.393	5.486	1.125 (1.316)	1.231	0.820
	Ratio	0.104 (0.197)	1.940	2.616	0.259 (0.249)	0.462	–1.094
Originality	Raw	7.806 (6.576)	1.531	2.735	3.625 (2.196)	0.696	–0.851
	Ratio	1.514 (0.426)	–0.725	–0.330	0.812 (0.368)	0.147	–1.474
Flexibility	Raw	5.111 (3.871)	1.232	1.528	3.083 (1.663)	0.315	–1.678
	Ratio	0.786 (0.248)	–1.006	–0.461	0.683 (0.236)	–0.292	–1.187
Elaboration	Raw	1.472 (1.104)	0.747	–0.326	1.417 (2.043)	2.125	3.609
	Ratio	0.466 (0.907)	3.245	9.798	0.277 (0.336)	1.884	2.929


### Prototypical Items

**FIGURE 1:**
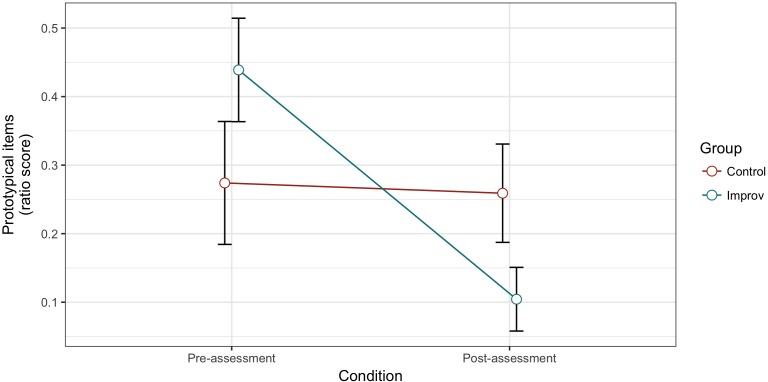
Alternative Uses Task (AUT) prototypical scores for pre- and post-test in the control and improv groups.

We found a main effect of Condition [*F*(1,28) = 9.713, *MSE* = 0.045, *p* = 0.004, $\eta_{p}^{2}$ = 0.258], with a higher prototypical items ratio in pre- [*M* = 0.373; *SD* = 0.323] than post-assessment [*M* = 0.166; *SD* = 0.228], but no Group effect [*F*(1,28) = 0.004, *MSE* = 0.103, *p* = 0.952, $\eta_{p}^{2}$ < 0.001]. There was a Group × Condition interaction [*F*(1,28) = 8.121, *MSE* = 0.045, *p* = 0.008, $\eta_{p}^{2}$ = 0.225], such as the improv group gave a smaller prototypical items ratio in the post-assessment compared to the control group while there was no difference in the pre-assessment (**Figure 1**).

### Fluency

The ANOVA revealed a main effect for Condition [*F*(1,28) = 4.590, *MSE* = 168.06, *p* = 0.041, $\eta_{p}^{2}$ = 0.141], with more items in the post- (*M* = 4.94; *SD* = 3.60), than in the pre-assessment (*M* = 2.94; *SD* = 2.03), but no Group effect [*F*(1,28) = 0.213, *MSE* = 421.94, *p* = 0.648, $\eta_{p}^{2}$ = 0.008] nor a Group × Condition interaction [*F*(1,28) = 1.196, *MSE* = 168.06, *p* = 0.283, $\eta_{p}^{2}$ = 0.041].

### Flexibility

**FIGURE 2:**
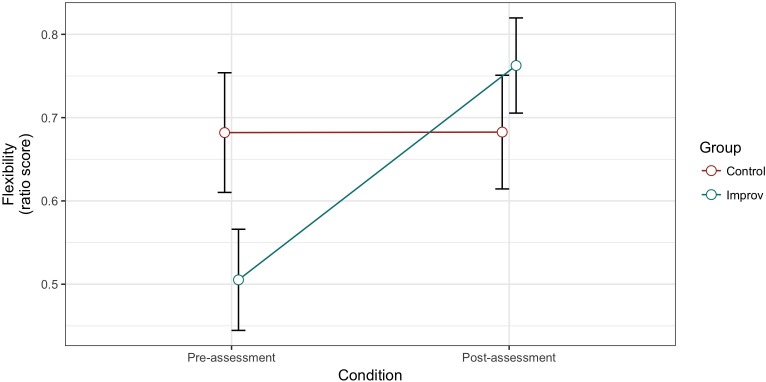
Alternative Uses Task (AUT) flexibility scores for pre- and post-test in the control and improv groups.

There was a main effect of Condition for flexibility [*F*(1,28) = 8.436, *MSE* = 0.041, *p* = 0.007, $\eta_{p}^{2}$ = 0.232], with higher ratio scores in the post-assessment (*M* = 0.745; *SD* = 0.245) than in the pre-assessment (*M* = 0.560; *SD* = 0.279) but no Group effect [*F*(1,28) = 0.412, *MSE* = 0.088, *p* = 0.526, $\eta_{p}^{2}$ = 0.014]. We found a Group × Condition interaction [*F*(1,28) = 8.371, *MSE* = 0.041, *p* = 0.0072, $\eta_{p}^{2}$ = 0.230], such as the improv group had a higher ratio scores for different categories in the post- than pre-assessment compared to the control group for which there was no difference (**Figure 2**).

### Originality

**FIGURE 3:**
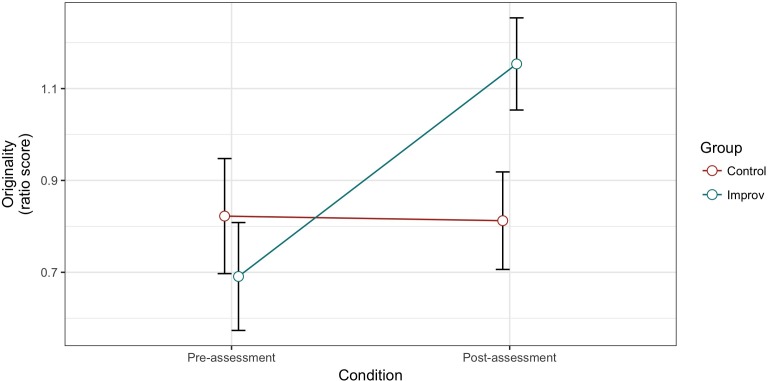
Alternative Uses Task (AUT) originality scores for pre- and post-test in the control and improv groups.

The ANOVA conducted on the originality ratio score showed a Condition effect [*F*(1,28) = 6.999, *MSE* = 0.105, *p =* 0.013, $\eta_{p}^{2}$ = 0.200], with better performance in the post-assessment, but no Group effect [*F*(1,28) = 0.561, *MSE* = 0.282, *p* = 0.460, $\eta_{p}^{2}$ = 0.020]. In the same way, we find a significant Group × Condition interaction [*F*(1,30) = 4.157, *MSE* = 0.131, *p* = 0.010, $\eta_{p}^{2}$ = 0.214], such as the Improv group outperformed the control group in the post-assessment condition while they did not differ in the pre-assessment (**Figure 3**).

### Elaboration

Using the ratio-ranked transformed data, there was no main effect of Group [*F*(1,28) = 0.341, *MSE* = 385.89, *p* = 0.564, $\eta_{p}^{2}$ = 0.012], Condition [*F*(1,28) = 1.903, *MSE* = 223.76, *p* = 0.179, $\eta_{p}^{2}$ = 0.064] nor Group × Condition interaction [*F*(1,28) = 1.962, *MSE* = 223.76, *p* = 0.172, $\eta_{p}^{2}$ = 0.065].

## Discussion

Our preliminary results confirm that improv can help enhance divergent thinking skills. We are the first to demonstrate, in our knowledge, that this effect exists with middle school learners. We discuss these results at theoretical (divergent thinking) and applied levels for pedagogical purposes.

### Divergent Thinking Evaluations

We found a divergent thinking improvement with better results after improv for originality and flexibility, but no difference for fluency nor elaboration. Our results are consistent with short improv sessions improvements with children (Sowden et al., 2015) and adults (Lewis and Lovatt, 2013).

The AUT scores showed differences between groups, conditions and within themselves. We showed improv benefits for Prototypical items, Originality and Flexibility. The improv group gave less prototypical items after the session than before. Prototypical items were not evoked in previous papers using AUT (Akbari Chermahini et al., 2012; Lewis and Lovatt, 2013), although it has been scored in similar task (Milgram et al., 1978; Harrington et al., 1983). In this experiment, we do not know if our population was particular and might not understand the instructions (although nothing indicated it), if Prototypical items were included in the fluency scores or simply not a specific issue in previous papers. While Flexibility performance was better for the control group in the pre-assessment, no difference can be found with the improv group after the improv sessions. The Flexibility ratio (Flexibility/Fluency) improved only for the improv group, supporting the binding process enhancement with improv training hypothesis. Consistently with previous findings (Lewis and Lovatt, 2013), the improv group participants were more original (Originality ratio score) than the control group after the training. In an embodied cognition perspective, we could hypothesize that repeated practice improves skills (Ito, 2008) and leads to better knowledge or faster access to it, thus to more different (original) items in the same amount of time. Koziol et al. (2012) argued that top down models cannot explain the continuously adaptive human behaviors and evolution leading directed to action control and not action *per se*. Future improv studies must focus on working memory, binding and control processes in general, beyond the classic executive functions definition (Miyake et al., 2000), with a double aim: a better understanding of improv impact and to develop a new way to investigate embodied cognition. The embodied cognition framework (Barsalou et al., 2003) is, in our opinion, the best to study improv. Barsalou (1999, p. 61) highlighted “the primary function of cognition is not to archive information but instead to prepare agents for situated action,” which is the core of improv.

On the null result side, there was no significant improvement for Fluency nor Elaboration. There is a Condition effect (post > pre) for the Fluency score, mostly due to the improv group improvement (+2.1 words on average) compared to the control group (+0.3 word on average), however, there was no Group × Condition interaction so we cannot conclude to an improv specific Fluency improvement here. This unexpected result is likely due to the important standard deviation in the improv group post-assessment, and might be different with a bigger sample. On the qualitative side, it highlights the heterogeneous profile and progress of teenagers and the need to address their psychological profile more extensively, including non-creative fluency (i.e., verbal category fluency test) and language skills in general. The absence of Elaboration difference was less of a surprise. Indeed, we can wonder if the classic Elaboration instructions are explicit enough. Nothing tells they have to elaborate their answer. In the school context, learners could give a simple short answer when nothing elaborate is asked, just because it was not asked. Learners, when they are in a group, tend to follow the exact instructions and their critical mind is not always at its best (Lacot et al., 2016). While the AUT is a widely used divergent thinking assessment, the instructions might need to be adapted, at least for children and teenagers.

Beyond confirming previous results, these results are consistent with children and adults’ positive impact of improv on divergent thinking. This suggests that the theater part of improv (also known as theatresports) has some importance in the divergent thinking enhancement, considering the difference with our sports control group. We believe further researches are needed to confirm our results in different ecological situations and population. Moreover, an in depth evaluation of improvisers’ cognitive and emotional profiles will be needed to understand which improv components help enhancing divergent thinking.

### Pedagogical Issues

While the research on improv might be trending in a few years, the educational impact has been the center of attention for decades. The French Education Ministry recently endorsed improv as a learning tool (Eduscol, 2017), consistent with recent projects involving with teachers and students (Hainselin et al., 2017b). In France, not only politics but the media focused their attention on improv, with a recent documentary *Liberté, Egalité, Improvisez* (Freedom, Equality, Improvise, based on France motto Liberty Equality, Fraternity), following teenagers enrolled in the *Trophée Culture et Diversité* (Culture and Diversity Trophy), a popular improv national tournament (Rotschild and Bergeon, 2014). Teenage improvisers, their teachers and parents praised for improv benefits in the classroom: academic performance, but also well-being, speaking in public skills and creativity. Robert Gravel, Canadian co-creator of the improvisational game, said: “The more rigid the structure, the more immovable the playing area is, the tougher the referee is, the more impeccable the MC is, then the more the madness is allowed within the game” (Lavergne and Gravel, 1993). Although, we obviously prefer divergent thinking over madness, the artistic point of view converges with scientific evidence. Gravel emphasized the need for a well-designed and prepared frame to enhance creation and richer play, which can be translated into cognitive flexibility and divergent thinking. Stanislavski (2013), famous Actor’s Studio inspirational model, also encouraged his actors and trainees to go beyond the classical “as if” to enhance personality, emotional and physical believability by demanding to embody the character.

These artistic anecdotes, combined with the growing literature on improv should encourage more researchers to run large-scale scientific research to assess the psychological impact of improv. A special focus is needed on teenagers, a very critical period in personality’s development and well-being (Moreira et al., 2015), notably in intellectually gifted (Villatte and de Léonardis, 2012). In this perspective, a study is being conducted to determine the impact of improv exercises on emotional aspects (i.e., anxiety, self-esteem and well-being) in intellectually gifted teenagers. The instantaneous adaptation to unexpected events, a core skill developed in improv, can benefit far beyond teenagers in their school environment. Previous research suggests improv can enhance adaptive behavior and metacognition (Biasutti, 2017). A recent paper highlights the benefits of applied improvisation for medical students’ training (Hoffmann-Longtin et al., 2017). One of the twelve tips “collaborate with experts in university theater departments, community theaters and/or improvisation ensembles” is particularly relevant for middle and high schools too, in collaboration with psychologists and psychology researchers. More particularly, the period of high performance, synchrony and enhanced sense of togetherness in improv, named “being in the zone” by Noy et al. (2015), is a very promising topic of research. In conclusion, improvisational theater (improv) helps teenagers improving divergent thinking. While future research is needed on this topic, we hope other specific psychological topics (i.e., memory, executive functions, anxiety, well-being, etc.) will be studied within the improv practice through lifespan.

## Author Contributions

MH designed the study and wrote the manuscript. AA ran the statistical analyses and drafted the manuscript. BB designed the study and drafted the manuscript.

## Conflict of Interest Statement

The authors declare that the research was conducted in the absence of any commercial or financial relationships that could be construed as a potential conflict of interest.
